# Hospital Festivals, Meetings, &c.

**Published:** 1894-04-28

**Authors:** 


### HOSPITAL FESTIVALS, MEETINGS, ETC.

### THE LIVINGSTONE MEMORIAL COTTAGE HOSPITAL, DARTFORD.

Saturday, the 20th inst., will long be remembered by the people of Dartford, Kent. For some years the inhabitants, who number nearly 12,000, have desired to have a hospital in order to obviate the sufferings and risks attending the removal of patients to the metropolitan hospitals.

Owing to the liberality of Mr. Burroughs, of Messrs. Burroughs and Wellcome, who has extensive works at Dartford, and who has given half the cost of the building (£1,000) now in course of erection, a cottage hospital containing sixteen beds will shortly be opened for the reception of patients. The friendly societies and labour organisations have taken great interest in the cottage hospital, and are working hard to raise the necessary funds to maintain it in efficiency. On Saturday the town was decorated, and the friendly societies marched in procession to the site, where Mr. H. M. Stanley was to lay the memorial stone. The weather was delightful, and Mr. and Mrs. Stanley could not have failed to be gratified by the enthusiasm of their reception.

Mr. Stanley, after declaring the stone duly laid, delivered a lengthy address, in which he touched on the chief features of Livingstone's life and work. His good-humoured indifference to the discomforts of his wandering life was, he said, as marked as his gracious bearing towards the negroes. His hands were as free from violence as his lips were without guile. His devotions were performed with exemplary regularity morning and evening; profanity was an abomination to him, vice in all forms was foreign to his nature, and they all knew what a long championship of hapless tribes he maintained against the marauding Arabs. He was the first Caucasian traveller who penetrated into the Chambezi and Lualaba Valleys; his was the first voice heard in behalf of the long-buried nations of the Upper Congo region, which denounced the slave-trader and his murderous acts; and in countries which until his time had been unknown to the most learned cartographer-he was the first to initiate the forbearance of the Founder of his religion, and to utter the sweet message of "Peace and good, will to men." His voice was hushed some twenty-one years ago near Lake Bangweolo, but the pale face and bowed head of this man, who had tasted misery to its lowest depths, was still remembered by the Manynema, by the people of Kazembe, and the inhabitants of Ujiji, and for years and years after he had departed for ever from their sight they wistfully expected his return to them, for they knew not that their gentle white friend was dead. Great indeed must be the nation that could produce from among its poorest class a hero of such broad benevolence and wide charity as Livingstone. If any came there that day with a vague idea that the man was a misanthrope from some sense of defeat, from some consciousness that he was not appreciated according to his deserts, such an estimate of Livingstone was wide of the truth. He had met few so quickly responsive to gaiety and the lighter moods who was more sociable, genial, tolerant, and humorous. They must think of him as of a contented soul who had yielded himself to a loving submission, and who laboured to the best of his means and ability, awaking to the toils of the day and resigning himself without the least misgiving to the rest of the night, believing that the effect of his self-renunciation would not be altogether barren.

Mr. Burroughs proposed, and Mr. Ernest Hart seconded, the vote of thanks to Mr. Stanley. Mr. Hart said Mr. Stanley had stood by Livingstone's side in the hour of his greatest need, and had brought him, through difficulties almost unheard of, the aid which had inspired him with new energies and enabled him to carry to a successful issue the labours of his later years. The medical profession welcomed Mr. Stanley that day as the one appropriate man to take the leading part in dedicating this cottage hospital to the great physician and African explorer.

Mr. Burroughs entertained the visitors, members of the committee, and leading inhabitants after the ceremony, and proposed a vote of thanks to the committee for their energetic labours in bringing the cottage hospital movement to its present state of success. The vote was seconded by Mr. Burdett, who emphasised the suitability of erecting a hospital as a memorial to a great explorer like Livingstone. If the memory of Livingstone was to be commemorated by the erection of a cottage hospital, Dartford, in the County of Kent, was certainly the one place where that building should stand. That was so because the County of Kent contained twenty hospitals, a number far in excess of those of any other county, showing that the inhabitants of Kent were keenly alive to the importance of the principle embodied in the cottage hospital idea, which was that every small community of intelligent people should give their medical practitioners the fullest opportunity of maintaining their knowledge and skill. Hospitals had their origin in, and depended for their maintenance upon the loving care and forethought which the hale had for the sick; hence a hospital was the very best kind of memorial to Livingstone, who as an explorer endured hardships and encountered risks in order to help the oppressed and barbarian races by introducing them to civilization and Christianity. Of course the advance of an explorer, representing, as he does, progress and development, must necessarily cause a certain amount of loss of life and suffering in the early days of his enterprise, still that suffering and loss of life were as nothing compared to the good which his labours brought to posterity, and might be likened to the action of a surgeon who removes a limb to save a useful life. It was meet, therefore, not only that Livingstone should be commemorated by the erection of a cottage hospital, but that Mr. Stanley, our greatest living explorer, should be the man to lay the memorial stone, and to utter the eloquent eulogy to which they had listened that day. All honour to Livingstone and Stanley, two men of whom all classes of Englishmen and many peoples had reason to be proud.

### LONDON LOCK HOSPITAL AND RESCUE HOME.

One of the most interesting events of the past few days was last Saturday's festival dinner, at the Hotel Metropole, of the London Lock Hospital and Rescue Home. This institution carries on what is practically a unique work in London, a work whose outcome and value is not, whatever may be the figures or facts quoted for or against it, to be estimated by a mere reference to the more apparent results in the present generation, and yet there is no other philanthropic agency at all akin which is more ignored or even, it may almost be said, contemned. One would have thought that with so many broadening influences around and among us, and the progress and diffusion of a more just, more rational, and more sympathetic method of regarding certain acute social questions, that an institution carrying on a well-regulated and efficacious work among fallen women, on lines which offer the greatest probability and hope of success, would have obtained at the hands of the public an ever-widening measure of practical recognition. Yet, unfortunately, this is hardly the case, for though the measure widens, it does so very slowly. This fact will be admitted by all who have worked in furtherance of the influence of the Lock Hospital, but yet one is pleased to observe no note of discouragement. The whole matter, even in this country, may have to be faced in a comparatively short time on lines which, altogether apart from their form, will have to be thorough, and the supporters of lock hospital work will be able to take special credit for pioneer labours in combating an evil which increases every day, a fact which was amply born out by Mr. Buxton Shillitoe in his response at the festival dinner to the toast of "The Medical Staff." The primary object of the holding of this dinner was to collect funds towards extinguishing an accumulated debt on the institution of £5,000. It is, of course, relatively a small sum, but that it is a thorough incubus is evident from the way in which it has clung to the finances of the hospital. In 1881 a determined effort reduced it to little over £2,000, but since then it has gone on varying, but never really permanently decreasing—the down grade fluctuations being due to the system of reckoning legacies as income—until it now reaches its present figures. Towards the desired liquidation a sum of £2,831 17s. 6d. was announced—satisfactory enough in itself, but noteworthy rather for the munificence of individuals—Baron de Hirsch, for instance, gave £500, and Lord Kinnaird £210—than for contributions denoting widespread interest and sympathy. The Duke of Connaught was unfortunately unable to keep his promise to occupy the chair. The Coburg wedding festivities detained him, an eventuality which had been very generally forecasted, but he sent a donation of £25. In his unavoidable absence, Lord Randolph Churchill generously, at very short notice, stepped into the breach, and he was supported by an audience which, if not numerically strong, for it did not much exceed one hundred, was at any rate cognisant of the merits of the work and enthusiastic in its support. Amongst those present were the Earl of Galloway, Lords Kinnaird, Falkland, and Teynham, the Hon. E. A. Fitzroy, Sir A. N. Agnew, Sir F. F. Buxton, Sir R. G. Head, Sir Lionel Darell, General Sir Reginald Gipps, Colonel Sir R. Nigel Kingscote, Sir E G. Jenkinson, Colonels Fraser, Cook, Arthur, and Harrington Stuart, the Revs. H. Wace, G. F. Pentecost, and H. Graham Thwaites, and Messrs. Lionel Darell, C. Hoare, George Herring, and Sydney Gedge. The medical profession was fairly well represented, a good proportion of the officers of the institution attending, while Dr. Robson Roose and Dr. Alfred Cooper were also present. The toast list was long, and on the whole the speeches were poor. Lord Randolph Churchill was by no means at his best, being at times practically inaudible; Lord Kinnaird's remarks were rather disconnected and curtailed owing to the lateness of the hour, while sundry speeches came so perilously near offending against those canons which endeavour to ensure that a non-political gathering shall be in tone actually what it nominally purports to be, that the Rev. G. F. Pentecost was enabled to make several humorous thrusts with a good deal of effect. It was, moreover, worthy of some notice that the various speakers preferred to deal with the general principle of lock hospital work rather than with the labours of the special institution for which they were pleading; but it may well be asked—is it safe to assume that guests at the festival dinner of a hospital know perfectly well even the main lines of the undertaking they are asked to support? To a certain extent they may, but their knowledge can always be supplemented, and a speech, if slightly statistical, need not necessarily be "dry." The outline of the hospital work for 1893, which has been issued by the management and of whose existence the speakers were aware, showed that the percentage of permanent reclamations is more than 25, though be it noted, for instance, that in the period mentioned 380 in all out of 739 patients who passed through the hospital were placed in homes or restored to their friends, 74 being received into the rescue institution in the Harrow Road. Although no direct reference was made in the speeches to the point, it is obvious to any inquirer that these inmates are precisely the class, and perhaps the only class—and it is a large one—whom rescue work of this kind can reach and reclaim. They seem, and probably are, of the domestic servant stamp, having doubtless in many cases gravitated from the country to the metropolis. Their training in the home, taking the form of laundry work under contract, needlework and kitchen work, is appropriate, and indirectly renders it possible that in the future this female branch of the undertaking may become like the male hospital and out-patients' department in Dean Street, Soho, self-supporting. At present, quite apart from the extinction of the debt, and despite sedulous and successful endeavours to reduce annual expenditure, a substantial increase in the annual subscriptions is necessary to put finance on a perfectly satisfactory footing, and to remove the reproach that some of the hospital wards are now closed.

Five preliminary toasts were disposed of on Saturday evening before the chief one was reached: "The Queen" and "The Prince and Princess of Wales and the other members of the Royal Family," proposed, of course, from the chair; "The Houses of Parliament," proposed by Mr. Gedge, ex-M.P. and Parliamentary candidate, and acknowledged by Lord Teynham and Lord R. Churchill; "The Clergy of all Denominations," proposed by the Chairman, and acknowledged by the Rev. H. Wace, Principal of King's College, and the Rev. G. F. Pentecost, both of whom emphasised the position of Christianity towards rescue work of the kind in question; and "The Army and Navy and Reserve Forces," proposed by that tried old volunteer Sir Fowell Buxton, and responded to by General Sir Reginald Gipps.

The Chairman then proposed "Prosperity to the London Lock Hospital and Rescue Home," stating he had four very good reasons for the special interest he took in the institution. In the first place, it was the only hospital of its kind in London, which fact, in view of the teeming population of the metropolis, made it deserving of the assistance of all wealthy men; in the second place, for various reasons, it did not receive the support to which it was entitled; the third reason was the immense amount of good that it had been effecting in spite of its limited resources, and the fourth was, he thought, the highest of all, viz., that wards were unoccupied on account of the lack of funds The object of the hospital was, in the first place, to try and get into its beneficent and healing hand some, at any rate, of the thousands of women suffering from the malady which the London Lock Hospital treated; and when anyone reflected on the effect of the evil, not only on the population of the present day, but on posterity, an evil which was growing worse and worse, could he hesitate to take the most practical and effective action to arrest the mischief and widen the extent of the work of the institution. But the area of the work of the hospital was not confined to the mere treatment of disease. The authorities tried, and tried with success, to reform at least some of the women who had been admitted and cured, and it would be difficult to exaggerate the importance of the results that had been achieved The management of the hospital had to fight against greater difficulties than, he thought, any institution had to contend with, and they were deserving of more public recognition than they had hitherto received. He thought the Lord Mayor and Corporation might be persuaded to hold some great meeting to be attended by the dignitaries of the Church, who ought to look upon the question as one of high morality, and to use their influence to secure for it public sympathy and support. He looked also to those occupying high positions in society, which brought with them great responsibilities, to interest themselves in the work, to turn their attention to human misfortune and frailty, and to do their utmost to alleviate it. (Cheers.)

Lord Kinnaird, who is a vice-president, and one of the two treasurers of the hospital, briefly responded, incidentally remarking that he did not believe that State aid, awarded in the shape of grants as at present to voluntary schools, would, in any way, interfere with the voluntary hospital system of which they were so justly proud, and which they wished to perpetuate. Sir A. N. Agnew proposed "The Medical Staff," who had also been eulogised by Lord Kinnaird, and to this the Senior Surgeon (Mr. Buxton Shillitoe) responded. In acknowledging the toast of his health, proposed by Lord Kinnaird, Lord Randolph closed the proceedings with a few more words of encouragement. The London Lock Hospital was, he said, perhaps the greatest work of charity now in London, and perhaps the best managed, and he hoped their example might have imitators working on the same lines the movement which the Harrow Road institution had initiated and carried on.

